# Cell jamming, stratification and p63 expression in cultivated human corneal epithelial cell sheets

**DOI:** 10.1038/s41598-020-64394-6

**Published:** 2020-06-09

**Authors:** Koichi Baba, Kei Sasaki, Mio Morita, Tomoyo Tanaka, Yosuke Teranishi, Takahiro Ogasawara, Yoshinori Oie, Izumi Kusumi, Masukazu Inoie, Ken-ichiro Hata, Andrew J. Quantock, Masahiro Kino-oka, Kohji Nishida

**Affiliations:** 1 Department of Ophthalmology, Graduate School of Medicine, Osaka University, 2-2 Yamadaoka, Suita, Osaka 565-0871 Japan; 2 Department of Biotechnology, Graduate School of Engineering, Osaka University, 2-2 Yamadaoka, Suita, Osaka 565-0871 Japan; 3 Global Center for Medical Engineering and Informatics, Osaka University, 2-2 Yamadaoka, Suita, Osaka 565-0871 Japan; 4 Japan Tissue Engineering Co., Ltd, 6-209-1 Miyakitadori, Gamagori, Aichi 443-0022 Japan; 5 Structural Biophysics Group, School of Optometry and Vision Sciences, Cardiff University, Maindy Road, Cardiff, Wales CF24 4HQ United Kingdom; 6 Integrated Frontier Research for Medical Science Division, Institute for Open and Transdisciplinary Research Initiatives (OTRI), Osaka University, 2-2 Yamadaoka, Suita, Osaka 565-0871 Japan

**Keywords:** Corneal diseases, Biomedical engineering

## Abstract

Corneal limbal epithelial stem cell transplantation using cultivated human corneal epithelial cell sheets has been used successfully to treat limbal stem cell deficiencies. Here we report an investigation into the quality of cultivated human corneal epithelial cell sheets using time-lapse imaging of the cell culture process every 20 minutes over 14 days to ascertain the level of cell jamming, a phenomenon in which cells become smaller, more rounded and less actively expansive. In parallel, we also assessed the expression of p63, an important corneal epithelial stem cell marker. The occurrence of cell jamming was variable and transient, but was invariably associated with a thickening and stratification of the cell sheet. p63 was present in all expanding cell sheets in the first 9 days of culture, but it’s presence did not always correlate with stratification of the cell sheet. Nor did p63 expression necessarily persist in stratified cell sheets. An assessment of cell jamming, therefore, can shed significant light on the quality and regenerative potential of cultivated human corneal epithelial cell sheets.

## Introduction

Corneal epithelial stem cells reside in the basal layer of the limbus, which is the transitional zone between the cornea and the bulbar conjunctiva^1–3^. Complete loss of limbal epithelial stem cells due to severe trauma (e.g., thermal and chemical burns) or eye disease (e.g., Stevens-Johnson syndrome or ocular pemphigoid) induces a corneal limbal stem cell deficiency (LCSD)^4,5^. In these situations, the function of renewing the corneal epithelium by generating transient amplifying cells that migrate from the limbus into the corneal basal layer is lost. Therefore, adjacent conjunctival tissue completely covers the cornea, leading to corneal vascularization and opacification with severe vision loss. As a therapy, limbal transplantation is usually performed, however, owing to a lack of donors it is difficult to conduct sufficient limbal transplantation for the number of waiting patients. In addition, postoperative outcomes of LSCD can be poor due to immune rejection^6–8^. To overcome these problems, cultivated limbal epithelial transplantation has become an alternative treatment^9–12^. It is a common recognition that cultivated limbal epithelial cells (LECs) should include a certain amount of corneal epithelium stem cells^13,14^. It is further appreciated that p63, a corneal epithelial stem cell maker, likely influences the proliferation and stratification of epithelial cells in emerging cultivated LEC sheets^15–19^. Therefore, knowledge of p63 in cultivated LEC sheets is perceived to be important from the standpoint of assessing the quality of the cell sheet.

Time-lapse imaging of cell cultures is an effective method that can noninvasively evaluate cell differentiation and proliferation in real time^20–22^ to produce the basis for a rational judgement for cell sheet quality evaluation and transplantation timing. In recent years particular attention has been paid to cell jamming whereby the collectively flow of cells is reduced, alongside increased cell proliferation, elevated cell density and crowding^23–28^. Cell jamming is considered to be directly related to the degree of stratification of cultivated LEC sheets, and we propose that an analysis of jamming can provide important clues in the understanding of epithelial cell proliferation, differentiation, and stratification. The purpose of this study was to evaluate p63 and cell jamming in *ex vivo* explanted cultivated LEC sheets to better understand the dynamics and likely final quality of the generated sheets.

## Results

### Variable and transient jamming of cells occurs in emerging cultivated LEC sheets

**Figure 1:**
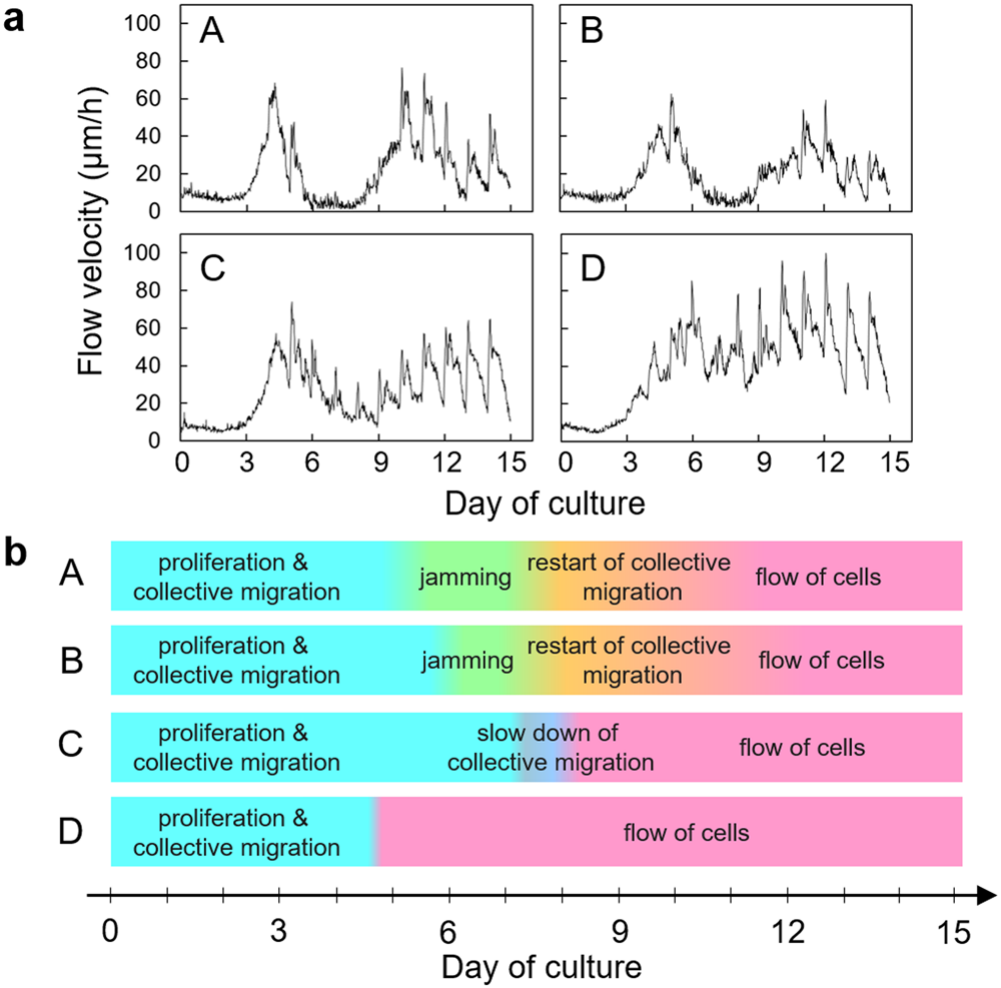
(**a**) PIV analysis and (**b**) cell behavior during culture. A: specimen 1, B: specimen 2, C: specimen 3, and D: specimen 4.

Time-lapse imaging of emerging cultivated LEC sheets revealed colonies of corneal epithelial cells forming between feeder cells between days 2 to 4 in culture, followed by corneal epithelial cells proliferating with collective migration (Supplementary Video S1). Subsequently, there was a tendency for the dynamic behavior of each of the four cultivated LEC sheets to differ (after day 5 of culture). In sheets 1 and 2, for example, the collective migration speed gradually slowed and became arrested, indicating that cell jamming had occurred (in specimen 1 from 5 to 7 days and in specimen 2 from 6 to 7 days). Cells then restarted migration followed by a characteristic non-jammed fluid-like collective flow after 10 days. In specimen 3, a full arrest of collective migration was not observed, although a temporary slowdown did occur from day 6 to day 8 of culture. In cultivated LEC sheet specimen 4, the collective migration of cells continued during the entire culture period with no evidence of cell jamming. To obtain quantitative data from the qualitative collective migration seen in the time-laps imaging, particle image velocimetry (PIV) was used (Fig. 1 and Supplementary Fig. S1). This confirmed what was seen in the time-lapse imaging for each of the four cultivated LEC sheets. Namely, that the collective migration became arrested around day 7 for specimens 1 and 2, indicative of cell jamming. A collective fluid-like flow of cells then occurred after day 8 of culture. For specimen 4, collective migration did not arrest during the whole culture period (i.e., cell jamming did not occur), whereas specimen 3 disclosed an intermediate behavior in which collective cell migration transiently slowed, but did not become fully jammed.

### Cell morphology changes during cultivated LEC sheet fabrication

**Figure 2:**
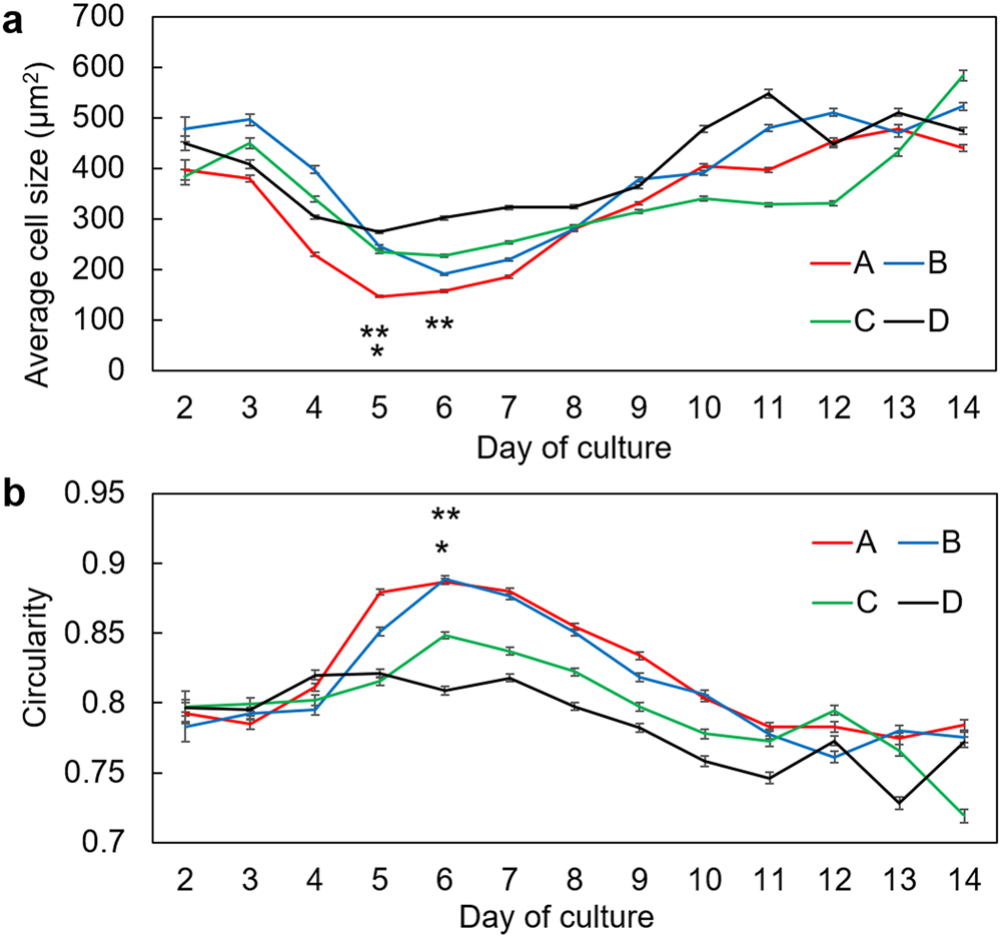
The relationship between culture day and (**a**) average cell area and (**b**) cell circularity. A: specimen 1, B: specimen 2, C: specimen 3, and D: specimen 4. Error bars indicate standard error. In the case of (**a**): for day 5 P < 0.0001 for between all specimens apart from specimen 2 vs specimen 3 (*P = 0.069), for day 6 **P < 0.0001 between each specimen. In the case of (**b**): **P < 0.0001 between each specimen except for specimen 1 vs specimen 2 (*P = 0.82).

The binarized data of the static pictures of time-lapse imaging was used to elucidate the change of mean cell size in the cultivated LEC sheets during culture. Common to all specimens, it was found that the mean cell size attained a transient minimum value around a third of the way through the cultivation period (Fig. 2a). The minimum mean cell size was 146.9 μm^2^ for specimen 1 (at day 5 of culture), 190.5 μm^2^ for specimen 2 (at day 6 of culture), 226.9 μm^2^ for specimen 3 (at day 6 of culturing), and 274.3 μm^2^ for specimen 4 (at day 5 of culture). In cultivated LEC sheets 1 and 2 the minimum mean cell size corresponded with the period of cell migration becoming temporarily arrested or jammed. Cell circularity reflects the morphology of a cell^29,30^; when the circularity index is high, the cell appears mostly round, whereas a low circularity is indicative of a less rounded, squamous cell. Our analysis showed that for each cultivated LEC sheet the maximum value of cell circularity occurred approximately a third of the way through the cultivation period, similar to when cells tended to be smallest. Values were 0.88 for specimen 1 (day 6), 0.88 for specimen 2 (day 6), 0.84 for specimen 3 (day 6) and 0.82 for specimen 4 (day 5), as shown in Fig. 2b.

### Colony forming assay of the cultivated LEC sheets

The viable cell density (×10^4^/cm^2^) of the cultivated LEC sheets was measured as 18.0, 19.8, 17.2, and 14.0 for specimens 1, 2, 3, and 4, respectively. Colony forming efficiency (CFE), which represents the proliferation and differentiation abilities of cells in the cultivated LEC sheets, was found to be 6.8%, 3.3%, 2.3%, and 3.1% for specimens 1, 2, 3, and 4, respectively. From these data a multiplicative proliferative capacity index for each cultivated LEC sheet (×10^4^/cm^2^) was calculated (which is reflective of the cell proliferation and cell differentiation capacity per unit area), and found to be 1.2, 0.7, 0.4, and 0.4 for specimens 1, 2, 3, and 4, respectively. The pertinent outcome of this analysis is that the proliferative capacity index of the cultivated LEC sheet specimens 1 and 2 is higher than that of specimens 3 and 4. All data are shown in Table 1.

**Table 1:** Colony forming assay and proliferative capacity index of cultivated LEC sheets.

Specimen No.	Age	Gender	Viable cell density (×10^4^/cm^2^)	CFE (%)	Proliferative capacity index of the cultivated LEC sheet (×10^4^/cm^2^)
1	60	M	18.0	6.8	1.2
2	55	F	19.8	3.3	0.7
3	49	M	17.2	2.3	0.4
4	57	F	14.0	3.1	0.4

### p63 immunofluorescence levels change during cultivated LEC sheet fabrication

**Figure 3:**
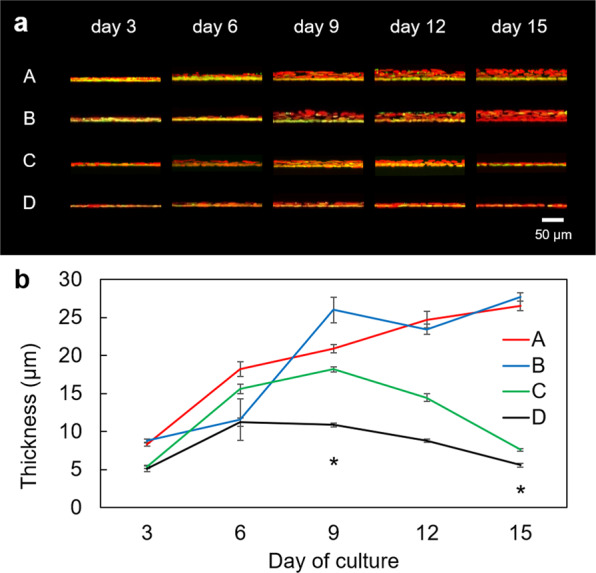
Time dependent change of cell sheet thickness. A: specimen 1, B: specimen 2, C: specimen 3, and D: specimen 4. (**a**) Confocal laser microscopic images of cell sheets: x-y layer. (**b**) Sheet thickness. Error bars indicate standard deviation. *P < 0.0001 between each sample for day 9 and day 15, respectively.

**Figure 4:**
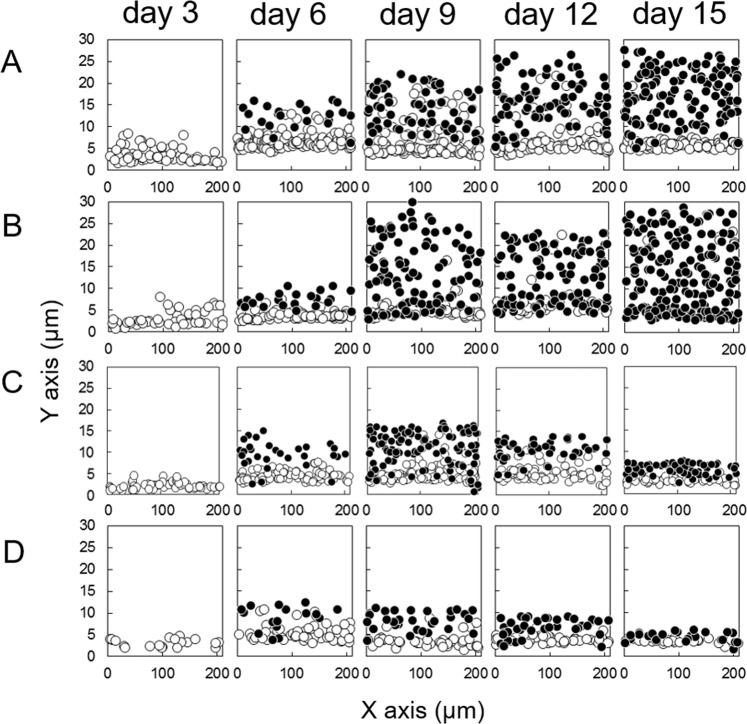
Presence and position of cells in cultivated LEC sheets. ● indicates nucleus. ○ indicates p63 positive nucleus.

In conventional evaluation methods of expanded corneal epithelial cells a cell sheet is chemically fixed and thin-sectioned, after which the presence of p63-positive cells is confirmed by immunostaining^15,31,32^. This can assess p63 expression in the thin-section, but cannot quantitatively evaluate the overall expression level of p63 inside the cell sheet. Therefore, we observed the cultivated LEC sheets three-dimensionally using confocal laser fluorescence microscopy (Fig. 3a), with p63 positive cells labelled. This data, described in Fig. 4 and Supplementary Table S1 and summarized in Supplementary Table S2, indicate that the p63 positive cell ratio in the basal cell layer remained constant and at a high level (95% or more) in cultivated LEC sheet specimen 1 from day 6 to 15 of culture. In specimen 2, after obtaining a peak value of p63 positive cells at day 6, a linear decrease was observed until day 15. For specimens 3 and 4 there was a trend of decreasing p63 positive cell numbers after their early peak values. As shown in Fig. 3b, the thickness of the cell sheet tended to increase in culture for specimens 1 and 2, suggesting that stratification of the cell sheets was occurring. In cultivated LEC sheet specimen 3 the thickness of the cell sheet increased until day 9 of culture, and thereafter decreased from day 12 to 15. In specimen 4, the thickness of the cell sheet did not change appreciably throughout the culture period; cell jamming, moreover, did not occur in this specimen.

## Discussion

The dynamic behavior of corneal epithelial cells in culture dishes underpins the formation of cultivated LEC sheets that can be used to recover vision in limbal stem cell deficient eyes. Here, we show that in the early stage after seeding, cells divide and grow and collectively migrate to occupy the relatively large growth space of the culture dish. However, when the colonies become bigger they tend to adhere each other and the cell size reduces with increasing cell density. Typically, at this time cell movement as whole slows gradually and can eventually arrest. This phenomenon is called cell jamming^26–28^, which in epithelial cells affects cell differentiation and stratification^27,28^. As summarized in Table 2, in cultivated LEC sheet specimens 1 and 2 the collective migration speed gradually decreased then arrested at around days 5 to 7. At this juncture, the mean cell size reached a minimum value and the cell circularity attained its maximum value, features of cell jamming. In cultivated LEC sheet specimen 4 on the other hand, while the mean cell size reached its minimum value and the cell circularity reached the maximum value at around the same time as in cultivated LEC sheet specimens 1 and 2, the collective migration continued during the whole culture period. Thus, cell jamming was judged not to have occurred. Specimen 3 seemed to take an intermediate path to specimens 1 and 2 and specimen 4. Namely, even though the collective migration speed slowed (day 6 to day 8) and was accompanied with the mean cell size and the cell circularity taking minimum and maximum values respectively, the migration did not completely arrest–i.e. cell jamming was not clearly identified.

**Table 2:** Summary of cell jamming, minimum cell size, maximum cell circularity, number of p63 positive basal cells, and stratification.

Specimen No.	Cell jamming	Min. cell size mean ± SE, μm^2^	Max. circularity mean ± SE	p63 positive cells in basal layer, ×10^5^ nuclei/cm^2^	Stratification mean ± SD, μm (day15)
1	observed (day 5–7)	146.9 ± 1.9 (day 5)	0.88 ± 0.002 (day 6)	2.71 (day 6)	good (26.5 ± 0.6)
2	observed (day 6–7)	190.5 ± 2.4 (day 6)	0.88 ± 0.002 (day 6)	1.68 (day 6)	good (27.7 ± 0.5)
3	insufficient (day 6–8)	226.9 ± 2.9 (day 6)	0.84 ± 0.002 (day 6)	2.40 (day 6)	weak (7.6 ± 0.1)
4	no	274.3 ± 3.3 (day 5)	0.82 ± 0.002 (day 5)	1.85 (day 6)	weak (5.6 ± 0.2)

Cell stratification appeared to occur in various forms throughout the whole cultivation period. For example, as summarized in Table 2, stratification was evident in cultivated LEC sheet specimens 1 and 2 when they underwent cell jamming. Sample 4, on the other hand, in which cell jamming did not occur displayed no apparent increase in sheet thickness or stratification. The stratification of cultivated LEC sheet specimen 3 – in which a slowdown of collective cell migration short of full cell jamming was seen – showed a transient increase in sheet thickness/stratification, with a diminishment after the flow of cells resumed. Based on these observations, we suggest a relationship between stratification of an expanding cultivated LEC sheet and cell jamming. Interestingly, we speculate that the transition from jamming to unjamming that was observed in specimens 1 and 2 after around 8 days in culture might relate to morphological changes in cells, as well as to overcrowding due to proliferation as has been suggested elsewhere^26,27,33–37^. To further understand jamming-unjamming phenomena, a kinetic interpretation focusing on the sheet formation and degradation rates due to turnover is probably warranted.

p63 positive cells appeared in high numbers in the basal layers of all four expanding cultivated LEC sheets in the early cultivation period. The pan-p63 4A4 monoclonal antibody (mAb) we used in the current study is not as specific as a maker of stem cells as the ΔNp63α isomer. But, we note that ΔNp63α can be detected by 4A4, as a pan-p63 mAb^38^. Indeed, the 4A4 antibody has been used to investigate holoclone formation (not transient amplifying cells, but stem cells) via rigorous clonal analysis^38^. Thus, we contend that p63 positive cells detected in this study using the pan-p63 4A4 mAb can be regarded as predominantly stem cells. LEC sheet specimens 1 and 2, in which cell jamming occurred, displayed different expression patterns of p63 in the basal layer after day 6. Namely, p63 positive cells were abundant in the basal layer up to 15 days of culture in specimen 1. On the other hand, p63 positive cells in specimen 2 lineally declined in number until day 15. In cultivated LEC sheets, the presence of p63 positive stem cells in the cell sheets is necessary to achieve and maintain good results clinically^13^. Judging from the experimental results presented here, however, it seems likely that confirming the existence of p63 positive cells in the basal layer is not sufficient in isolation to estimate the quality of a cultivated LEC sheet with regards to its stratification (Table 2). Cell sheets which do not undergo cell jamming, we note, exhibit poor stratification even if there are adequate numbers of p63 positive cells in basal layer of the cell sheet.

The proliferative capacity index, described earlier, can reflect the degree of cell proliferation and cell differentiation per unit area in LEC sheet, and the likely appropriateness for use in surgery. In this study, LEC sheet specimens 1 and 2 had a higher proliferative capacity index than specimens 3 and 4 (Table 1). Stratification with cell proliferation was observed in LEC sheet specimens 1 and 2 during the culture period, but not in specimens 3 and 4 (Supplementary Table S1 and Fig. 3). Accordingly, from this small sample size, the proliferative capacity index and occurrence of cell jamming appear to be correlated, thus cell jamming could be regarded as a new measure that can determine the degree of cell proliferation and cell differentiation in cultivated LEC sheets. The colony forming assay which forms part of the proliferative capacity index calculation is a destructive evaluation method which takes about two weeks to conduct. An analysis of cell jamming, on the other hand, is non-destructive and can be performed in real-time during culture, which has a clear advantage over measurements of colony forming assays. Thus, we consider that an analysis of cell jamming is a novel and effective non-invasive assessment index for the evaluation of cultivated human corneal epithelial cell sheets.

## Methods

### Cultivated LEC sheet production

In line with the tenets of the Declaration of Helsinki, four corneas were obtained for research purposes from the Rocky Mountain Lions Eye Bank (Aurora, CO, USA). Donors were aged between 49 and 60 years old at the time of death. Specimens of limbal tissue were dissected and treated with trypsin at 37 °C, after which epithelial cells were collected and co-cultured (at 37 °C in 5% CO_2_) with 3T3-J2 feeder cells which had been deprived of their proliferative ability by x-ray irradiation. The culture medium used was DMEM and Ham’s F12 media (2:1 mixture) containing FCS (10%), insulin (5 mg/ml), adenine (0.18 mM), hydrocortisone (0.4 mg/ml), cholera toxin (0.1 nM), triiodothyronine (2 nM), glutamine (4 mM), and penicillinstreptomycin (50 IU/ml), epidermal growth factor (10 ng/ml)^39^. Corneal epithelial cells which reached subconfluent in primary culture were treated with trypsin at 37 °C followed by collected and then cryopreserved. The cells, which had been cryopreserved, were defrosted and co-cultured with 3T3-J2 feeder cells as described above, but using thermo-responsive culture dishes (UpCell 3.5 cm dish, Cell Seed, Japan), for up to 15 days. Cells cultured for 3, 6, 9, 12, and 15 days were used for immunocytochemistry, while cells cultured in parallel on other dishes were examined by time-lapse imaging from days 2 to 14.

### Colony forming assay of cultivated LEC sheets and calculation of a proliferative capacity index

To evaluate the colony forming efficiency in the 8 day cultivated LEC sheets, cells taken from trypsinized LEC sheets at this time were plated onto 3T3 feeder layers and cultivated. Colonies were fixed 10 to 14 days later, stained with rhodamine B and scored under a stereomicroscope. Colony forming efficiency is expressed as the ratio of the number colonies to the number of inoculated cells. The number of viable cells per unit area of the LEC sheet was calculated by dividing the number of viable cells after trypsinization by the area of the culture dish (8.8 cm^2^). A proliferative capacity index of each cultivated LEC sheets (×10^4^/cm^2^), which reflects cell proliferation and differentiation per unit area, was calculated by multiplying the viable cell density of the LEC sheet (×10^4^/cm^2^) and the colony forming efficiency (%).

### Immunostaining of p63

Cultured cells in their dishes were washed with PBS three times, then fixed with 4% PFA for 20 min. Three washes with PBS were followed by the addition of 2 ml of 0.5% Triton X-100 (MP Biomedicals 807426) to the dish for 20 min. The Triton X-100 solution was then removed and PBS (2 ml) added for 2 min. This was repeated three times, after which blocking solution Image-IT FX signal enhancer (Thermo Fisher Scientific, Japan) was added to the dish (0.5 ml for 30 min). The blocking solution was then removed and 0.5 ml of mouse anti-p63 monoclonal antibody (4A4, Nichirei Bioscience Inc., Japan) was added to the dish, which was kept at 4 °C overnight in the dark. The solution was removed and the specimens washed with PBST (0.05% Tween20 in PBS; 2 ml) three times for 2 mins each wash. The secondary antibody PBST solution (0.5 ml) was added to the dish which was kept at room temperature for 30 min in the dark. The secondary antibody PBST solution contained 1,000 times diluted Alexa Fluor 488 F(ab’) fragment of goat anti-mouse IgG(H + L) (Life Technologies A11017) and 1,000 times diluted solution of 1 mM Hoechst. The solution was removed then washed with PBST (2 ml) three times for 2 mins each wash. After removing the solution, Fluorescent Mounting Medium (Dako S3023) was added, and a coverslip glued in place. The circumference of each dish was removed using a laser cutter prior to observation by confocal laser fluorescence microscopy.

### Time-lapse imaging

For time-lapse imaging, the phase contrast image (phase difference × 4 objective lens) of all four specimens was measured every 20 min using a Biostudio-T microscope (Nikon Engineering, Kanagawa, Japan) throughout the whole cultivation period from 2–14 days.

### Analysis of corneal epithelial cells by PIV

Particle image velocimetry (PIV) was used to determine the flow velocity of the collective migration of cells in culture from the time-lapse imaging data. The PIV plugin of Image J (NIH data) was used for this analysis, with the size of the tracking target inspection window (interrogation window) set at 64 × 64 pixels (84.8 × 84.8 μm^2^). The mean of the central value of the velocity (scalar quantity) which was a plurality measurement was regarded as the representative flow velocity of the specimen (n = 3 wells for each specimen). The observation area was set at the center of each well.

### Confocal microscopy imaging of p63

Immunofluorescence imaging of LEC sheets was conducted using a confocal laser scanning microscope (FV-1000; Olympus) through a 60 × objective lens. Images were acquired at 256 × 256 pixels (212 × 212 μm^2^) every 0.45 μm through the thickness of the LEC sheet. The center of gravity positions of the corneal epithelial cell nucleus and the p63 (+) cell nucleus were measured using Image-Pro Plus version 6.0 software (Media Cybernetics, Silver Spring, MD, USA, http://www.mediacy.com/imageproplus). The basal layer was determined as previously described^40^. Briefly, the threshold (mean + 2 SD) of the gravity position of the measured cell nucleus was determined where the region of the cell nucleus did not overlap in the height direction at day 6. Then the cell in which the gravity position of the nucleus was lower than the threshold was used as the basal layer. The ranges of the basal layers of specimens 1, 2, 3 and 4 were 7.55 μm, 6.26 μm, 7.43 μm and 7.59 μm, respectively. The position of the bottom surface was determined by autofluorescence from the culture dish. Based on each picture, the distribution of p63 positive cells and non-positive cells in the x-y-z axis space (maximum space 212 × 212 × 212 μm^3^) was projected onto the x-y plane (Fig. 4). This means that the graphs in Fig. 4 do not represent a single section, but rather a “fly-through” in the z-direction of a 3D dataset, 212 μm in depth. The counted number of cells is averaged (n = 3 for each point) then shown in the table (Supplementary Table S1). The thickness of the cell sheet was estimated using the center of gravity position of the nuclei for each specimen.

### Generation of 14-day time-lapse video

The original static images of each of the four specimens taken every 20 mins from 2–14 days were saved in TIF format and incorporated into Power Point. To map the cells in the static image, each cell was line-enclosed manually after which it was automatically blacked out on Power Point. When the morphology of the cell was unclear, the cell was not blacked out. Subsequently, binarization of cells, as well as an analysis of their mean size and circularity were carried out using Image J.

### Statistical analysis

For statistical analysis, Tukey HSD was performed after an un-paired one-way analysis of variance (AOVA) of the data presented in Figs. 2 and 3. Tukey HSD was performed after an un-paired repeated one-way analysis of variance (ANOVA) of the data presented in supplementary figure S1. When a p-value was less than 5.0% (p < 5.0%), we regarded it as significant. For statistical analysis, soft wear of KaleidaGraph (Synergy Software) was used.

## Supplementary information

Supplementary Information.

Supplementary Information2.
