# Supplementary material for: Cell jamming, stratification and p63 expression in cultivated human corneal epithelial cell sheets
Source: Sci Rep. 2020 Jun 9;10:9282. doi: 10.1038/s41598-020-64394-6 (PMC7283219; doi:10.1038/s41598-020-64394-6)
Supplement: Supplementary file 1 — Supplementary Information. [file 41598_2020_64394_MOESM1_ESM.pdf]

Electronic Supplementary Information for:

# Cell jamming, stratification and p63 expression in cultivated human corneal epithelial cell sheets

Koichi Baba[1], Kei Sasaki[2,3], Mio Morita[1], Tomoyo Tanaka[4], Yosuke Teranishi[4], Takahiro Ogasawara[4], Yoshinori Oie[1], Izumi Kusumi[1], Masukazu Inoie[4], Kenichiro Hata[4], Andrew J Quantock[5], Masahiro Kino-oka[2], Kohji Nishida[1,6*]

[1] Osaka University Graduate School of Medicine, Department of Ophthalmology, 2-2 Yamadaoka, Suita, Osaka 565-0871 Japan

[2] Department of Biotechnology, Graduate School of Engineering, Osaka University, 2-2 Yamadaoka, Suita, Osaka, 565-0871, Japan

[3] Global Center for Medical Engineering and Informatics, Osaka University, 2-2 Yamadaoka, Suita, Osaka, 565-0871, Japan

[4] Japan Tissue Engineering Co., Ltd, 6-209-1 Miyakitadori, Gamagori, Aichi 443-0022, Japan

[5] Structural Biophysics Group, School of Optometry and Vision Sciences, Cardiff University, Maindy Road, Cardiff, Wales, CF24 4HQ, United Kingdom

[6] Integrated Frontier Research for Medical Science Division, Institute for Open and Transdisciplinary Research Initiatives (OTRI), Osaka University Director

* Corresponding author: knishida@ophthal.med.osaka-u.ac.jp

## Supplementary Figure, Tables, and Video

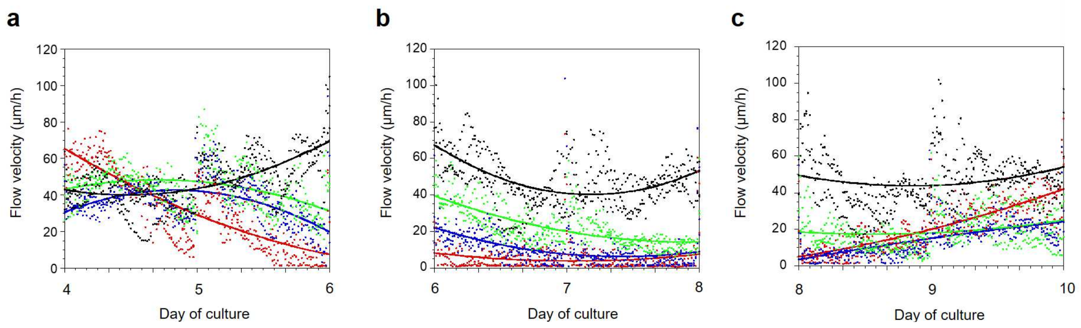


**Supplementary Figure S1.** PIV analysis. Specimen 1 (red), specimen 2 (blue), specimen 3 (green), and specimen 4 (black). (a) 4 to 6 days of culture, (b) 6 to 8 days of culture, (c) 8 to 10 days of culture. Significant difference; (a, b) P <0.0001 between each specimen. (c) P <0.0001 between each specimen except specimen 1 vs 3 (P = 0.018)

Table. Number of nuclei (x10³ nuclei/cm²)

| | | day 3 | | day 6 | | day 9 | | day 12 | | day 15 | |
|---|---|---|---|---|---|---|---|---|---|---|---|
| | | Basal | Suprabasal | Basal | Suprabasal | Basal | Suprabasal | Basal | Suprabasal | Basal | Suprabasal |
| Specimen 1 | total | 142 | 3 | 280 | 100 | 293 | 323 | 158 | 213 | 165 | 284 |
| | p63+ | 142 | 3 | 271 | 50 | 284 | 114 | 153 | 47 | 161 | 42 |
| | % of p63 | **100** | 100 | **96.6** | 50.4 | **97.0** | 35.1 | **96.7** | 22.3 | **97.3** | 14.6 |
| Specimen 2 | total | 108 | 16 | 185 | 39 | 160 | 175 | 127 | 208 | 151 | 262 |
| | p63+ | 108 | 16 | 168 | 4 | 131 | 29 | 49 | 36 | 0 | 13 |
| | % of p63 | **100** | 100 | **90.4** | 9.6 | **81.9** | 16.5 | **38.6** | 17.4 | **0.5** | 4.8 |
| Specimen 3 | total | 129 | 0 | 263 | 77 | 238 | 209 | 230 | 158 | 265 | 16 |
| | p63+ | 129 | 0 | 240 | 13 | 179 | 25 | 193 | 30 | 148 | 4 |
| | % of p63 | **100** | ND | **91.2** | 16.3 | **75.1** | 12.1 | **83.9** | 19.2 | **55.7** | 27.3 |
| Specimen 4 | total | 45 | 0 | 194 | 32 | 114 | 45 | 166 | 30 | 114 | 0 |
| | p63+ | 45 | 0 | 185 | 16 | 99 | 13 | 121 | 1 | 66 | 0 |
| | % of p63 | **100** | ND | **95.8** | 48.8 | **87.6** | 27.9 | **72.8** | 5.0 | **57.8** | ND |

**Supplementary Table S1.** Quantitative analysis of number of nuclei ($\times 10^3$ nuclei/cm$^2$) in cell sheets analyzed by confocal laser microscopy (n = 3 for each).

Table. Percentage of p63 positive cells in basal layer (%)

| No./days | day 6 | day 9 | day 12 | day 15 | average |
|---|---|---|---|---|---|
| Specimen 1 | 96.6 | 97.0 | 96.7 | 97.3 | 96.8 |
| Specimen 2 | 90.4 | 81.9 | 38.6 | 0.5 | 52.9 |
| Specimen 3 | 91.2 | 75.1 | 83.9 | 55.7 | 76.5 |
| Specimen 4 | 95.8 | 87.6 | 72.8 | 57.8 | 78.5 |

**Supplementary Table S2.** p63 positive cells in basal layer of the cell sheet.

## Caption for supplementary video (provided in separate file):

**Supplementary Video S1.** Time lapse imaging of human corneal limbal epithelial cells in culture. A: specimen 1, B: specimen 2, C: specimen 3, and D: specimen 4. The bar indicates 500 μm.
